# Amyloid‐dependent and amyloid‐independent effects of Tau in individuals without dementia

**DOI:** 10.1002/acn3.51457

**Published:** 2021-10-07

**Authors:** Joseph Therriault, Tharick A. Pascoal, Marcus Sefranek, Sulantha Mathotaarachchi, Andrea L. Benedet, Mira Chamoun, Firoza Z. Lussier, Cécile Tissot, Bruna Bellaver, Pamela S. Lukasewicz, Eduardo R. Zimmer, Paramita Saha‐Chaudhuri, Serge Gauthier, Pedro Rosa‐Neto, for the Alzheimer's Disease Neuroimaging Initiative

**Affiliations:** ^1^ Translational Neuroimaging Laboratory The McGill University Research Centre for Studies in Aging Montreal Canada; ^2^ Montreal Neurological Institute Montreal Canada; ^3^ Douglas Hospital Research Centre Le Centre intégré universitaire de santé et de services sociaux (CIUSSS) de l'Ouest‐de‐l'Île‐de‐Montréal McGill University Montreal Canada; ^4^ Graduate Program in Biological Sciences: Biochemistry Universidade Federal do Rio Grande do Sul Porto Alegre Brazil; ^5^ Department of Pharmacology Graduate Program in Biological Sciences: Pharmacology and Therapeutics Universidade Federal do Rio Grande do Sul Porto Alegre Brazil; ^6^ Department of Mathematics and Statistics University of Vermont Vermont USA; ^7^ Department of Neurology and Neurosurgery McGill University Montreal Canada

## Abstract

**Objective:**

To investigate the relationship between the topography of amyloid‐β plaques, tau neurofibrillary tangles, and the overlap between the two, with cognitive dysfunction in individuals without dementia.

**Methods:**

We evaluated 154 individuals who were assessed with amyloid‐β PET with [^18^F]AZD4694, tau‐PET with [^18^F]MK6240, structural MRI, and neuropsychological testing. We also evaluated an independent cohort of 240 individuals who were assessed with amyloid‐β PET with [^18^F]Florbetapir, tau‐PET with [^18^F]Flortaucipir, structural MRI, and neuropsychological testing. Using the VoxelStats toolbox, we conducted voxel‐wise linear regressions between amyloid‐PET, tau‐PET, and their interaction with cognitive function, correcting for age, sex, and years of education.

**Results:**

In both cohorts, we observed that tau‐PET standardized uptake value ratio in medial temporal lobes was associated with clinical dementia rating Sum of Boxes (CDR‐SoB) scores independently of local amyloid‐PET uptake (FWE corrected at *p* < 0.001). We also observed in both cohorts that in regions of the neocortex, associations between neocortical tau‐PET and clinical function were dependent on local amyloid‐PET (FWE corrected at *p* < 0.001).

**Interpretation:**

In medial temporal brain regions, characterized by the accumulation of tau pathology in the absence of amyloid‐β, tau had direct associations with cognitive dysfunction. In brain regions characterized by the accumulation of both amyloid‐β and tau pathologies such as the posterior cingulate and medial frontal cortices, tau’s relationship with cognitive dysfunction was dependent on local amyloid‐β concentrations. Our results provide evidence that amyloid‐β in Alzheimer’s disease influences cognition by potentiating the deleterious effects of tau pathology.

## Introduction

The role of amyloid‐β in the cognitive dysfunction which characterizes Alzheimer’s disease (AD) has been a matter of extensive debate.^1^ Current versions of the amyloid cascade hypothesis stipulate that amyloid‐β is a disease trigger for numerous other pathophysiological processes leading to tau hyperphosphorylation, neuroinflammation, and neurodegeneration,^2^, ^3^ eventually resulting in cognitive dysfunction. A large body of literature has identified multiple neurotoxic roles for amyloid‐β, including synaptic dysfunction^4^ and synapse loss.^5^, ^6^ However, the frequent appearance of elevated amyloid‐β in individuals without detectable cognitive impairment^7^ challenges the purported relationship between amyloid‐β and neural dysfunction suggested by experimental studies.

Recent human imaging studies suggest that aggregation of tau into neurofibrillary tangles, rather than amyloid‐β, is closely linked with clinical status, with tau‐PET patterns recapitulating regional glucose hypometabolism^8^ and domain‐specific cognitive dysfunction.^8^, ^9^ These studies, coupled with the observation of amyloid‐β reaching a plateau early in the disease^10^, ^11^ have led to an emerging framework in which AD is characterized by amyloid‐dependent and amyloid‐independent phases.^12^, ^13^

Postmortem^14^ and in vivo^15^, ^16^ studies have documented a characteristic sequential pattern of tau aggregation beginning in the medial temporal lobes, eventually spreading to multisensory association areas and subsequently primary sensory areas of the neocortex. Amyloid‐β aggregation, on the other hand, is characterized by early neocortical aggregation in regions such as the posterior cingulate, precuneus, and medial prefrontal cortices.^17^, ^18^ Building on reports of heightened toxicity in the presence of both amyloid‐β and tau pathologies, the colocalization of amyloid‐β and tau in neocortical regions highlights the possibility that the neurotoxic effects of tau may be potentiated by local amyloid‐β in a region‐dependent manner.

Here, we test the hypothesis that amyloid‐β potentiates the effects of tau pathology on clinical function in AD. Based on the reported topographical patterns of amyloid‐β and tau pathologies, we hypothesize that tau in the medial temporal lobes will be directly associated with cognitive dysfunction, while neocortical tau’s effects will be potentiated by local amyloid‐β. We measured amyloid‐β and tau pathology with PET in two independent cohorts of cognitively unimpaired (CU) elderly and individuals with mild cognitive impairment (MCI). Using a novel analytical framework, we tested whether associations between tau pathology and clinical function are dependent on local amyloid‐β concentrations.

## Materials and Methods

### Participants

#### TRIAD

The Translational Biomarkers in Aging and Dementia (TRIAD) cohort aims at describing biomarker trajectories and interactions as drivers of dementia.^19^ TRIAD was launched in 2017 as part of the McGill Centre for Studies in Aging. We assessed cognitively normal individuals (*n* = 124) as well as individuals with MCI (*n* = 50) who underwent amyloid‐β PET with [^18^F]AZD4694, tau‐PET with [^18^F]MK6240, structural MRI, and genotyping for *APOEϵ4*. All subjects had detailed clinical assessments including mini‐mental state examination (MMSE), clinical dementia rating (CDR), and cerebrovascular disease risk with the Hachinski Ischemic scale.^20^ Cognitively normal controls had a CDR of 0 and individuals with MCI had a CDR of 0.5. Inclusion criteria for all subjects are the ability to speak English or French, good general health (no diseases expected to interfere with study participation over time), absence of claustrophobia, and adequate visual and auditory capacities to follow neuropsychological evaluation. This study’s protocol was approved by McGill University’s Institutional Review Board and informed written consent was obtained from each subject. There was no attempt to match cases between cohorts.

#### ADNI

In this study, we also assessed cognitively normal individuals (*n* = 157) as well as individual with amnestic MCI (*n* = 83) individuals from Alzheimer’s Disease Neuroimaging Initiative (ADNI) cohort who underwent amyloid‐β PET with [^18^F]Florbetapir, tau‐PET with [^18^F]Flortaucipir, structural MRI, and genotyping for *APOEϵ4*. Cognitively normal controls had a CDR of 0, MCI subjects had a CDR of 0.5. The ADNI study was approved by the Institutional Review boards of all of the participating institutions. Informed written consent was obtained from all participants at each site. Full information regarding the ADNI inclusion and exclusion criteria can be accessed at http://adni.loni.usc.edu/.

### Genetic analyses

#### TRIAD

Determination of *APOE* genotypes for subjects recruited at McGill was performed using the polymerase chain reaction amplification technique, followed by restriction enzyme digestion, standard gel resolution, and visualization processes. Full details of this procedure can be found elsewhere.^21^

#### ADNI

Determination of *APOE* genotypes for ADNI subjects took place at the University of Pennsylvania Alzheimer’s Disease Biomarker Laboratory. Complete details of genetic methods employed in ADNI can be accessed at http://adni.loni.usc.edu/data‐samples/clinical‐data/.

### PET image acquisition and processing

#### TRIAD

All subjects had a T1‐weighted MRI which was used for coregistration. PET imaging acquired in the TRIAD cohort has been described previously.^22^ [^18^F]MK6240 images were acquired 90–110 min postinjection and scans were reconstructed with the OSEM algorithm on a 4D volume with four frames (4 × 300 sec).^23^ [^18^F]AZD4694 images were acquired 40–70 min postinjection and scans were reconstructed with the OSEM algorithm on a 4D volume with three frames (3 × 600 sec).^24^ The reconstruction algorithm is a 3D ordinary Poisson ordered subset expectation maximization (OP‐OSEM)^25^ with point spread function^26^ modeling, using 16 subsets and 10 iterations. Immediately following each PET acquisition, a 6‐min transmission scan was conducted with a rotating ^137^Cs point source for attenuation correction. Additionally, the images underwent correction for dead time, decay, and random and scattered coincidences. T1‐weighted images were nonuniformity and field‐distortion corrected and processed using an in‐house pipeline. Then, PET images were automatically registered to the T1‐weighted image space, and the T1‐weighted images were linearly and nonlinearly registered to the ADNI template space. Subsequently, a PET nonlinear registration was performed using the linear and nonlinear transformations from the T1‐weighted image to the ADNI space and the PET to T1‐weighted image registration, using ANTs. The PET images were spatially smoothed to achieve a final resolution of 8 mm full‐width at half maximum. [^18^F]MK6240 standardized uptake value ratio (SUVR) maps were generated using the inferior cerebellar grey matter as a reference region and [^18^F]AZD4694 SUVR maps were generated using the cerebellar grey matter as a reference region.^27^ A global [^18^F]AZD4694 SUVR value was estimated for each participant by averaging the SUVR from the precuneus, prefrontal, orbitofrontal, parietal, temporal, anterior, and posterior cingulate cortices; all voxels were weighted equally.^28^

#### ADNI

Full information regarding acquisition and preprocessing of PET data in ADNI is provided at http://adni.loni.usc.edu/data‐samples/pet/. Preprocessed PET images downloaded from ADNI underwent spatial normalization to the ADNI standardized space using the transformations of PET native to MRI native space and MRI native to the ADNI space. [^18^F]Flortaucipir (also known as [^18^F]T807 and/or [^18^F]AV1451) SUVR maps were generated using the inferior cerebellar grey matter as a reference region^29^ and [^18^F]Florbetapir SUVR maps were generated using the cerebellar grey matter as a reference region. A global [^18^F]Florbetapir SUVR value was estimated for each participant by averaging the SUVR from the precuneus, prefrontal, orbitofrontal, parietal, temporal, anterior, and posterior cingulate cortices.^28^

### Statistical analyses

Two independent samples were investigated: (1) the TRIAD cohort assessed with [^18^F]MK6240 and [^18^F]AZD4694 (2) an ADNI cohort assessed with [^18^F]Flortaucipir and [^18^F]Florbetapir. The primary outcome measure of the study was clinical function as measured by the Clinical Dementia Rating Sum of Boxes (CDR‐SoB), in which higher scores indicate more impaired functioning. In each cohort, we tested whether amyloid‐β potentiates relationships between tau pathology and cognitive dysfunction.

Baseline demographic and clinical data were assessed using *t* tests and χ^2^ tests. Neuroimaging analyses were carried out using the VoxelStats toolbox (https://github.com/sulantha2006/VoxelStats), a MATLAB‐based analytical framework that allows for the execution of multimodal voxel‐wise neuroimaging analyses.^30^ Other statistical analyses were performed using the R Statistical Software Package version 3.5.3 (http://www.r‐project.org/). Amyloid‐PET and tau‐PET images were centered on the mean of each cohort in order to improve coefficient interpretability numerical stability for estimation associated with multicollinearity.^31^ Given the large number of covariates in the statistical models, model diagnostics were carried out using the car package in R to determine the presence of multicollinearity.

In the TRIAD cohort, the voxel‐based model outlined below was built to test whether main effects and interactive effects of [^18^F]AZD4694 SUVR and [^18^F]MK6240 SUVR on CDR‐SoB. The model was also adjusted for sex, years of education, and age. Statistical parametric maps were corrected for multiple comparisons using Random Field Theory^32^ with a voxel threshold of *p* < 0.001 and a cluster threshold of *p* < 0.05. In every brain voxel, the model was of the form:

$$\text{CDR SoB}=\beta_{0}+\beta_{1}\left[{\;}^{18}F\right]\text{AZD}4694\;\text{SUVR}+\beta_{2}\left(\left[{\;}^{18}F\right]\text{MK}6240\;\text{SUVR}\right)+\beta_{3}\left(\left[{\;}^{18}F\right]\text{AZD}4694\;\text{SUVR}\times \left[{\;}^{18}F\right]\text{MK}6240\;\text{SUVR}\right)+\beta_{4}\left(\text{Age}\right)+\beta_{5}\left(\text{Sex}\right)+\beta_{6}\left(\text{Education, years}\right)+\in$$

Next, we tested the same hypothesis in the ADNI database, examining main and interactive effects of [^18^F]Florbetapir and [^18^F]Flortaucipir on CDR‐SoB. This model was also adjusted for sex, years of education, and age. Statistical parametric maps were corrected for multiple comparisons using Random Field Theory^32^ with voxel threshold of *p* < 0.001 and a cluster threshold of *p* < 0.05. In every brain voxel, the model was of the form:

$$\text{CDR SoB}=\beta_{0}+\beta_{1}\left(\left[{\;}^{18}F\right]\text{Florbetapir SUVR}\right)+\beta_{2}\left(\left[{\;}^{18}F\right]\text{Flortaucipir SUVR}\right)+\beta_{3}\left(\left[{\;}^{18}F\right]\text{Florbetapir SUVR}\times \left[{\;}^{18}F\right]\text{Flortaucipir SUVR}\right)+\beta_{4}\left(\text{Age}\right)+\beta_{5}\left(\text{Sex}\right)+\beta_{6}\left(\text{Education, years}\right)+\in$$

In each cohort, we further tested the adequacy of the models the interaction terms using an analysis of variance, comparing the interaction model with each reduced model testing amyloid‐PET plus tau‐PET, as well as the inclusion of the interaction term.^33^ Statistical analyses were repeated using MMSE score and rey auditory verbal learning test (RAVLT) delayed score as outcome measures. Statistical analyses were also repeated using global measures of amyloid‐PET.

## Results

Demographic and clinical information for both samples examined in this study are summarized in Table 1. Demographic comparisons between cohorts are reported in Table S1. CU individuals in TRIAD had higher baseline CDR‐SoB scores than did with CU individuals in ADNI. Variance inflation factors (VIFs) for all variables were between 1 and 2, indicating that problematic levels of multicollinearity are not present in our analyses.^34^

**Table 1:** Demographic and key characteristics of the samples.

	CN	MCI	*p* value
(A) TRIAD			
No.	138	26	**—**
Age, year, mean (SD)	68.32 (11.54)	74.4 (5.45)	0.007
Male, no. (%)	53 (38)	13 (50)	0.3
Education, year, mean (SD)	15.17 (3.77)	14.36 (3.79)	0.84
*APOE ϵ4 heterozygous,* %	43 (31)	9 (34)	0.21
*APOE ϵ4 homozygous,* %	1 (0.7)	1 (4)	0.17
MMSE, mean (SD)	29.05 (1.25)	27.13 (2.39)	<0.0001
CDR SoB, mean (SD)	0.18 (0.45)	1.47 (1.23)	<0.0001
[^18^F]AZD4694 SUVR, (SD)	1.48 (0.42)	1.86 (0.54)	0.0001
(B) ADNI			
No.	157	83	**—**
Age, year, mean (SD)	70.98 (5.91)	70.57 (7.09)	0.63
Male, no. (%)	71 (45)	49 (59)	0.04
Education, year, mean (SD)	16.65 (2.5)	15.84 (2.85)	0.02
*APOE ϵ4 heterozygous,* %	44 (28)	13 (15.6)	0.08
*APOE ϵ4 homozygous,* %	5 (3.1)	11 (13.3)	0.008
MMSE, mean (SD)	28.97 (1.33)	28.05 (2.15)	<0.0001
CDR SoB, mean (SD)	0.009 (0.51)	1.46 (0.93)	<0.0001
[^18^F]Florbetapir SUVR, (SD)	1.2 (0.22)	1.26 (0.29)	0.07

*p* values reported are for comparisons to cognitively normal subjects. *p* values indicate values assessed with independent samples *t*‐tests for each variable except sex and APOE *ϵ4* status, where contingency χ^2^ tests were performed. MMSE, Mini‐Mental State Examination; CDR SoB, Clinical Dementia Rating Sum of Boxes; SUVR, standardized uptake value ratio; CN, cognitively normal; MCI, mild cognitive impairment; TRIAD, Translational Biomarkers in Aging and Dementia; ADNI, Alzheimer’s Disease Neuroimaging Initiative.

**Figure 1:**
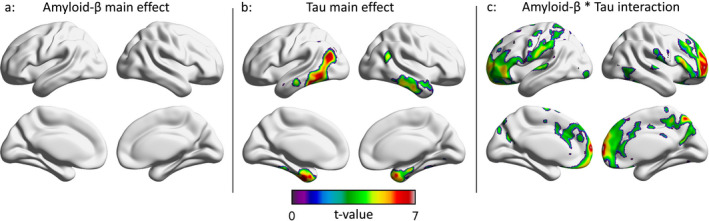
Regional associations between amyloid‐β, tau, and clinical function in the TRIAD cohort. T‐statistical parametric maps were corrected for multiple comparisons using a random field theory voxel threshold of *p* < 0.001 and a cluster threshold of *p* < 0.05, overlaid on a reference template. Age, sex, and years of education were used as covariates the model. (A) There was no significant main effect of [^18^F]AZD4694‐PET SUVR on clinical function across the brain. (B) There were main effects of [^18^F]MK6240‐PET SUVR on CDR‐SoB in the temporo occipital, basolateral temporal, and medial temporal lobes. (C) Interactions between [^18^F]AZD4694‐PET SUVR and [^18^F]MK6240‐PET SUVR on CDR‐SoB were observed in the posterior cingulate, precuneus, medial prefrontal, orbitofrontal, and dorsolateral prefrontal cortices. CDR SoB, Clinical Dementia Rating Sum of Boxes; SUVR, standardized uptake value ratio; TRIAD, Translational Biomarkers in Aging and Dementia.

In the TRIAD cohort, no significant relationships between amyloid‐PET SUVR and clinical function were observed (Fig. 1A). Higher tau‐PET SUVR in medial temporal and inferior temporal cortices was associated with impaired clinical function (Fig. 1B). Voxel‐level interactions between continuous measures of amyloid‐PET and continuous measures of tau‐PET in the medial prefrontal, dorsolateral prefrontal, anterior cingulate, posterior cingulate, and precuneus cortices were associated with impaired clinical function (Fig. 1C). In regions where the interaction term was significant, the main effects of amyloid‐β and tau‐PET SUVR on impaired clinical status were negligible. Results using MMSE and RAVLT delayed scores in the TRIAD cohort are displayed in Figures S1A–C, S2A–C. Scatter plots of the distribution of [^18^F]AZD4694, [^18^F]MK6240, and CDR‐SoB for the TRIAD cohort are reported in Figures S3, S4.

**Figure 2:**
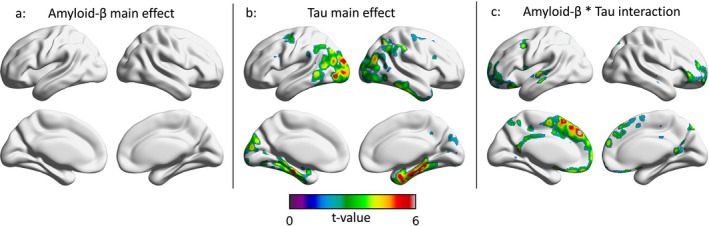
Regional associations between amyloid‐β, tau, and clinical function in the ADNI cohort. T‐statistical parametric maps were corrected for multiple comparisons using a random field theory voxel threshold of *p* < 0.001 and a cluster threshold of *p* < 0.05, overlaid on a reference template. Age, sex, and years of education were used as covariates the model. (A) There was no significant main effect of [^18^F]Florbetapir‐PET SUVR on clinical function across the brain. (B) inferior parietal, basolateral temporal, and medial temporal lobes, as well as the occipital cortex. (C) Interactions between [^18^F]Florbetapir‐PET SUVR and [^18^F]Flortaucipir‐PET SUVR on CDR‐SoB were observed in the posterior cingulate, precuneus, anterior cingulate, medial prefrontal, and dorsolateral prefrontal cortices. CDR SoB, Clinical Dementia Rating Sum of Boxes; SUVR, standardized uptake value ratio; ADNI, Alzheimer’s Disease Neuroimaging Initiative.

In the ADNI cohort, no significant relationships between amyloid‐PET SUVR and clinical function were observed (Fig. 2A). Higher tau‐PET SUVR in medial temporal, inferior temporal, and occipital cortices was associated with impaired clinical function (Fig. 2B). Voxel‐level interactions between continuous measures of amyloid‐PET and continuous measures of tau‐PET in the medial prefrontal, orbitofrontal, superior frontal, anterior cingulate, posterior cingulate, and precuneus cortices were associated with impaired clinical function (Fig. 2C). Similar to the results obtained in the TRIAD cohort, the main effects of amyloid‐β and tau‐PET SUVR on impaired clinical status were negligible in regions where the interaction term was significant. Results using MMSE and RAVLT delayed scores in the ADNI cohort are displayed in Figures S1D‐F, S2D‐F. Scatter plots of the distribution of [^18^F]Florbetapir, [^18^F]Flortaucipir, and CDR‐SoB for the ADNI cohort are reported in Figures S3, S4.

**Figure 3:**
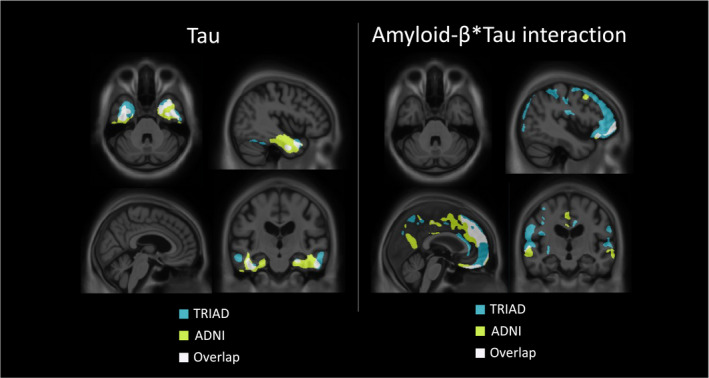
Topographical overlap between TRIAD and ADNI cohorts of tau and amyloid‐β × tau interaction effects. Left: overlap between the topography of main effects of tau‐PET on CDR‐SoB in the TRIAD and ADNI cohorts. Right: overlap between the topography of amyloid‐β × tau interaction effects on CDR‐SoB in the TRIAD and ADNI cohorts. CDR SoB, Clinical Dementia Rating Sum of Boxes; TRIAD, Translational Biomarkers in Aging and Dementia; ADNI, Alzheimer’s Disease Neuroimaging Initiative.

Analysis of variance supported that in both cohorts, the model including the interaction term best described the relationship between amyloid‐PET, tau‐PET, and clinical status (Table 2). Summaries of statistical outcomes from both TRIAD and ADNI cohorts are reported in Table 3. A schematic of the topographical overlap of results obtained from TRIAD and ADNI cohorts is displayed in Figure 3.

**Table 2:** Analysis of variance results.

	AIC	Adjusted *R* ^2^	*F*‐statistic	*p* value
(A) TRIAD				
Additive model	900	0.25	10.9	< 0.0001
Interactive model	892	0.36	14.77	< 0.0001
(B) ADNI				
Additive model	941	0.18	13.01	< 0.0001
Interactive model	931	0.23	14.06	< 0.0001

In the TRIAD cohort, the interactive model better explained cognitive decline as opposed to the additive model (*p* = 0.002). Similarly, in the ADNI cohort, the interactive model better explained cognitive decline as opposed to the additive model (*p* = 0.0005). TRIAD, Translational Biomarkers in Aging and Dementia; ADNI, Alzheimer’s Disease Neuroimaging Initiative.

**Table 3:** Main and interactive effects of amyloid‐PET and Tau‐PET uptake on CDR Sum of Boxes.

Brain region	Amyloid‐PET main effect estimate (SE)	Tau‐PET main effect estimate (SE)	Amyloid‐PET × Tau‐PET interaction effect estimate (SE)
(A) TRIAD Tau‐PET cohort			
Posterior cingulate	−3.66 (1.04)	5.47 (2.44)	3.53 (0.91)
Precuneus	−5.64 (1.1)	8.69 (2.19)	5.08 (0.93)
Anterior cingulate	−2.77 (0.88)	5.43 (2.16)	2.98 (0.83)
Medial prefrontal	−2.78 (0.69)	3.66 (1.41)	2.67 (0.59)
Dorsolateral prefrontal	−3.19 (0.77)	4.41 (1.38)	2.83 (0.59)
Orbitofrontal	−3.93 (0.80)	4.89 (1.31)	3.62 (0.64)
Medial temporal	0.89 (0.4)	2.19 (0.26)	−2.95 (0.69)
Lateral temporal	0.83 (0.29)	1.86 (0.24)	−1.25 (0.32)
(B) ADNI Tau‐PET cohort			
Posterior cingulate	−6.92 (1.48)	−7.43 (1.89)	7.12 (1.25)
Lateral temporal	−10.57 (1.83)	−7.14 (1.95)	11.51 (1.86)
Orbitofrontal	−9.02 (1.85)	−10.32 (2.25)	11.73 (1.89)
Medial prefrontal	−5.70 (1.55)	−7.56 (2.02)	8.00 (1.61)
Medial temporal	−1.87 (2.88)	16.618 (2.71)	−8.97 (2.12)
Basal temporal	1.95 (1.65)	4.68 (0.55)	−1.839 (1.32)
Occipital	5.39 (1.78)	10.39 (2.39)	−5.39 (1.6)

This table reports main and interactive effects of amyloid‐PET and Tau‐PET on CDR Sum of Boxes. The amyloid‐PET and Tau‐PET interaction effect estimate is higher than the sum of the individual effects, indicating the presence of a synergistic interaction. TRIAD, Translational Biomarkers in Aging and Dementia; ADNI, Alzheimer’s Disease Neuroimaging Initiative; CDR, clinical dementia rating.

## Discussion

This study supports the hypothesis that amyloid‐β contributes to clinical symptoms by potentiating tau‐dependent cognitive dysfunction. In two cohorts, we observed that while amyloid‐PET SUVR at the voxel level was not associated with cognitive dysfunction, tau‐PET SUVR in the medial temporal lobes had a direct relationship with cognitive dysfunction. Moreover, in regions such as the medial prefrontal cortex, anterior cingulate, posterior cingulate, and precuneus cortices, amyloid‐PET levels potentiated tau’s relationship with clinical function. Taken together, these findings build on recent tau‐PET studies in humans^35^ to suggest that amyloid‐β is associated with cognition by potentiating tau‐dependent cognitive dysfunction.

In our study, the amyloid‐independent effects of tau pathology on cognitive dysfunction were largely confined to the medial temporal lobes. Evidence from autopsy studies^36^ as well as in vivo studies^15^ suggests that the medial temporal lobes are a site of early tau aggregation. Crucially, the medial temporal regions are also regions in which amyloid‐β plaques aggregate later in the course of AD, and in lower concentrations.^14^, ^37^ Correspondingly, it is plausible that the amyloid‐independent effects of tau pathology on cognitive dysfunction are related to the lower concentrations of amyloid‐β in these regions. Further supporting this idea is the finding that the amyloid‐dependent effects of tau pathology were observed in regions of the brain’s default mode network, characterized by significant and early amyloid‐β accumulation.^17^, ^38^, ^39^ This study extends previous research conducted using CSF concentrations of phosphorylated tau which reported interactions between amyloid‐β and tau concentrations associated with cerebral metabolic dysfunction as well as longitudinal cognitive dysfunction.^33^, ^40^ Building on these studies, our study leverages the topographical information garnered by PET imaging to provide evidence of specific regional patterns of amyloid‐dependent and amyloid‐independent associations of tau with cognitive dysfunction, in which both the quantity and localization of amyloid‐β modulate the effects of tau.

Contemporary versions of the amyloid cascade hypothesis of AD posit that amyloid‐β leads to AD through initiating a series of events including tau hyperphosphorylation, neuroinflammation, and neurodegeneration, among other events, eventually leading to cognitive dysfunction.^3^ Our study contributes to this model by providing evidence that in addition to acting as a disease trigger, amyloid‐β contributes to cognitive impairment through local interactions with tau pathology. The finding of deleterious interaction between amyloid‐β and tau pathologies in humans is in line with studies from experimental animals in which molecular interactions between amyloid‐β and tau peptides lead to synaptic^41^ and neural circuit dysfunction.^42^ Furthermore, cell culture studies have provided evidence that amyloid‐tau interactions are associated with deficits in axonal transport^43^ and exacerbate neuronal death.^44^ Moreover, our results are in line with accepted AD biomarker models which suggest that amyloidosis alone is not sufficient for cognitive dysfunction.^45^, ^46^ However, our results contribute to this framework by providing evidence that amyloid‐β may be more than a disease trigger: amyloid‐β contributes to cognitive dysfunction through potentiating tau’s effects on cognitive dysfunction.

Tau accumulation in the medial temporal cortex was associated with cognitive dysfunction. These results are in line with studies of individuals with primary age‐related tauopathy (PART), characterized by medial temporal tau accumulation accompanied by mild cognitive dysfunction, in the absence of amyloid‐β.^47^ Individuals with PART, display slower rates of cognitive dysfunction^48^ and rarely progress to dementia.^47^ Taken together, these findings further support the role of amyloid‐β in the cognitive dysfunction that characterizes AD.

From a therapeutic perspective, our findings highlight the possibility that anti‐amyloid therapies may be beneficial to slow cognitive dysfunction in the symptomatic phase of AD by reducing amyloid‐β potentiating of tau’s pathological effects. However, based on the results presented in this study, anti‐amyloid therapies may be less effective at slowing the progression of memory dysfunction mediated by medial temporal cortical regions. More studies directly assessing longitudinal cognitive decline, as well as memory decline specifically, are needed to further support this notion. As disease‐modifying trials continue to shift toward earlier disease phases,^49^ targeting amyloid‐β before the appearance of tau pathology remains a promising strategy.^50^ Finally, it is also important to consider the role of neurodegeneration in cognitive decline. Because neurodegeneration is considered to at least partially mediate associations between AD pathology and cognition,^51^ future longitudinal studies including neurodegeneration biomarkers are needed.

While the majority of associations between PET measures and cognitive dysfunction were observed in both TRIAD and ADNI cohorts, some results were only observed in one cohort. For example, tau accumulation in occipital cortices was associated with cognitive dysfunction in ADNI, but not in TRIAD. Furthermore, in some areas such as the precuneus and lateral temporal cortices, little physical overlap was observed, but significant clusters were observed in the same region in both cohorts. It is conceivable that these differences in results are attributable to differences in the individuals enrolled in each study. More studies probing the specific nature of cognitive dysfunction may find that tau accumulation in these regions are associated with cognitive dysfunction in specific domains.^8^

Methodological limitations should be considered when interpreting this study. The first is that the study is not designed to uncover biological mechanisms for the potentiation of tau’s effects by amyloid‐β. Moreover, TRIAD and ADNI are both research cohorts consisting of highly motivated individuals to participate in AD research and may not reflect the general population. Third, the spatial resolution of PET imaging places limitations on the capacity to describe molecular interactions; the colocalization of elevated amyloid‐PET and tau‐PET uptake within a voxel is not the same as identifying amyloid‐tau interactions at the molecular level. However, our study builds on previous preclinical studies^41^ to identify amyloid‐β’s potentiation of tau in living humans. Fourth, it is also plausible that cytoarchitectural differences of the medial temporal lobes,^52^ rather than concentrations of amyloid‐β, are related to differential vulnerability to amyloid‐dependent versus amyloid‐independent effects of tau reported in this study. Future experiments using preclinical models may shed light on this question. Methodological advantages of the study include large samples, replication in an independent multicenter study despite in baseline CDR‐SoB scores, the use of continuous variables and replication of tau‐PET results with both first‐generation and second‐generation radiotracers.

## Conflict of Interest

None of the authors declare relevant conflict of interest.

## Author Contributions

JT, TAP, SM, and PRN: Study concept and design, image processing and analysis, figure, and manuscript draft. ALB, MS, MC, FL, and CT: Image processing and analysis, figure, and manuscript draft. GM, PSC, JP, and SG: Study concept and design, and critical review of manuscript for intellectual content.

## Supporting information


**Figure S1.** Regional associations between amyloid‐β, tau, and MMSE in the TRIAD and ADNI cohorts.

**Figure S2.** Regional associations between amyloid‐β, tau, and RAVLT delayed recall in the TRIAD and ADNI cohorts.

**Figure S3.** 3D scatter plot of the distribution of amyloid‐PET and tau‐PET on CDR Sum of Boxes.

**Figure S4.** 3D scatter plot of main effects of medial temporal tau‐PET on CDR Sum of Boxes.

**Table S1.** Between‐sample demographic comparisons.
