# Supplementary figures and images for: Amyloid‐dependent and amyloid‐independent effects of Tau in individuals without dementia
Source: Ann Clin Transl Neurol. 2021 Oct 7;8(10):2083–92. doi: 10.1002/acn3.51457 (PMC8528464; doi:10.1002/acn3.51457)

Amyloid- $\beta$  main effect

Tau main effect

Amyloid- $\beta$  \* tau interaction

TRIAD (n = 154)

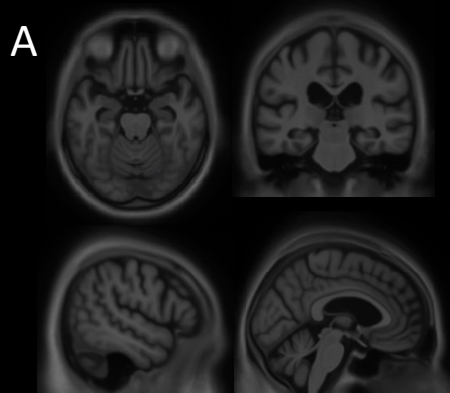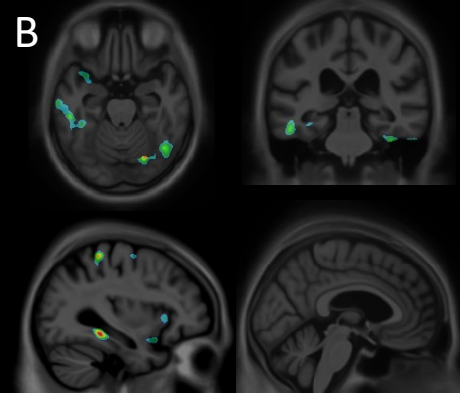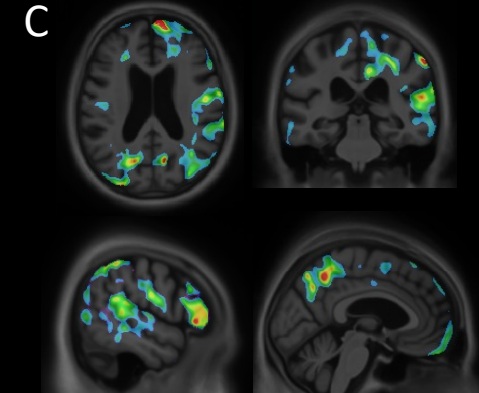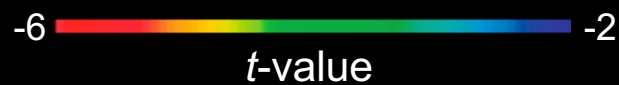

ADNI (n = 240)

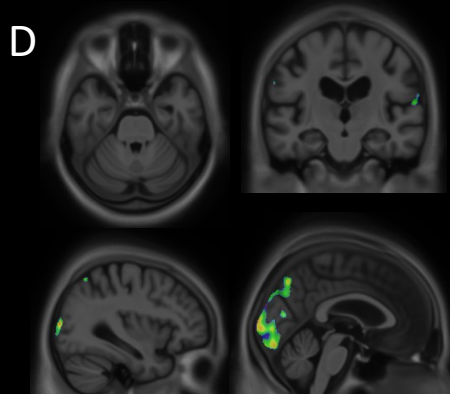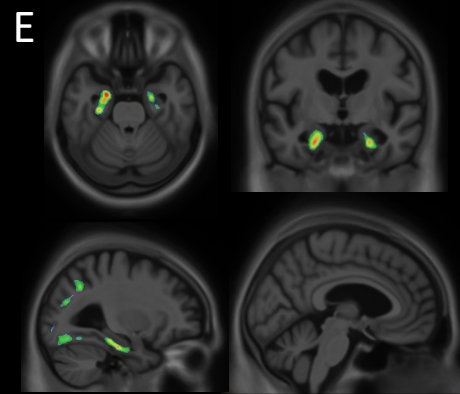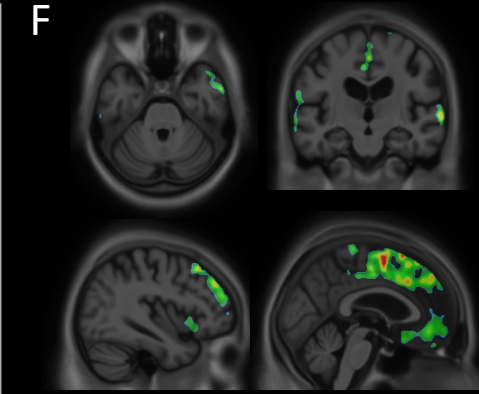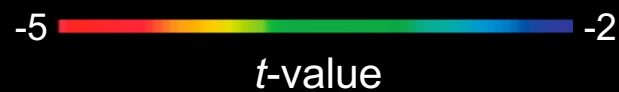

Supplement: Supplementary file 1 — Figure S1. Regional associations between amyloid‐β, tau, and MMSE in the TRIAD and ADNI cohorts. [file ACN3-8-2083-s004.pdf]

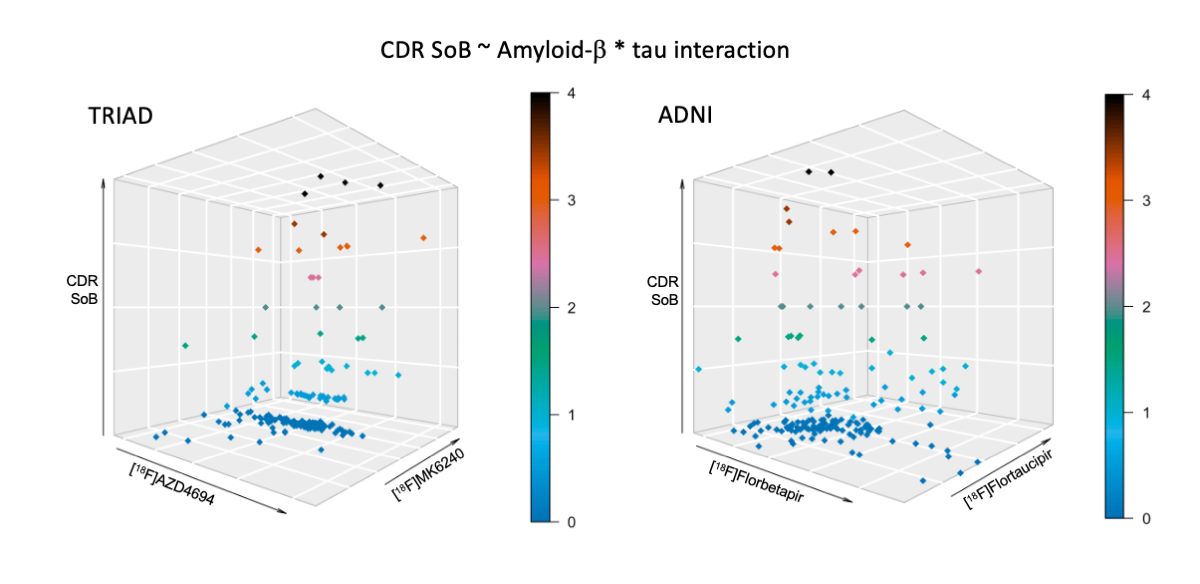

Supplement: Supplementary file 3 — Figure S3. 3D scatter plot of the distribution of amyloid‐PET and tau‐PET on CDR Sum of Boxes. [file ACN3-8-2083-s005.png]

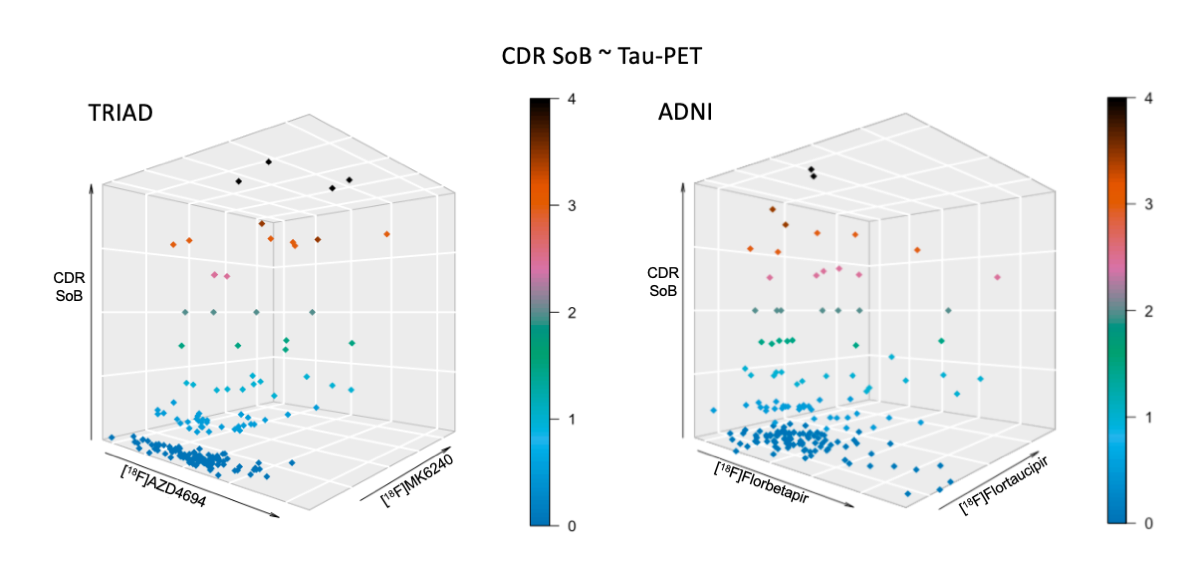

Supplement: Supplementary file 4 — Figure S4. 3D scatter plot of main effects of medial temporal tau‐PET on CDR Sum of Boxes. [file ACN3-8-2083-s002.png]
