# Supplementary material for: Amyloid‐dependent and amyloid‐independent effects of Tau in individuals without dementia
Source: Ann Clin Transl Neurol. 2021 Oct 7;8(10):2083–92. doi: 10.1002/acn3.51457 (PMC8528464; doi:10.1002/acn3.51457)
Supplement: Supplementary file 2 — Figure S2. Regional associations between amyloid‐β, tau, and RAVLT delayed recall in the TRIAD and ADNI cohorts. [file ACN3-8-2083-s001.pdf]

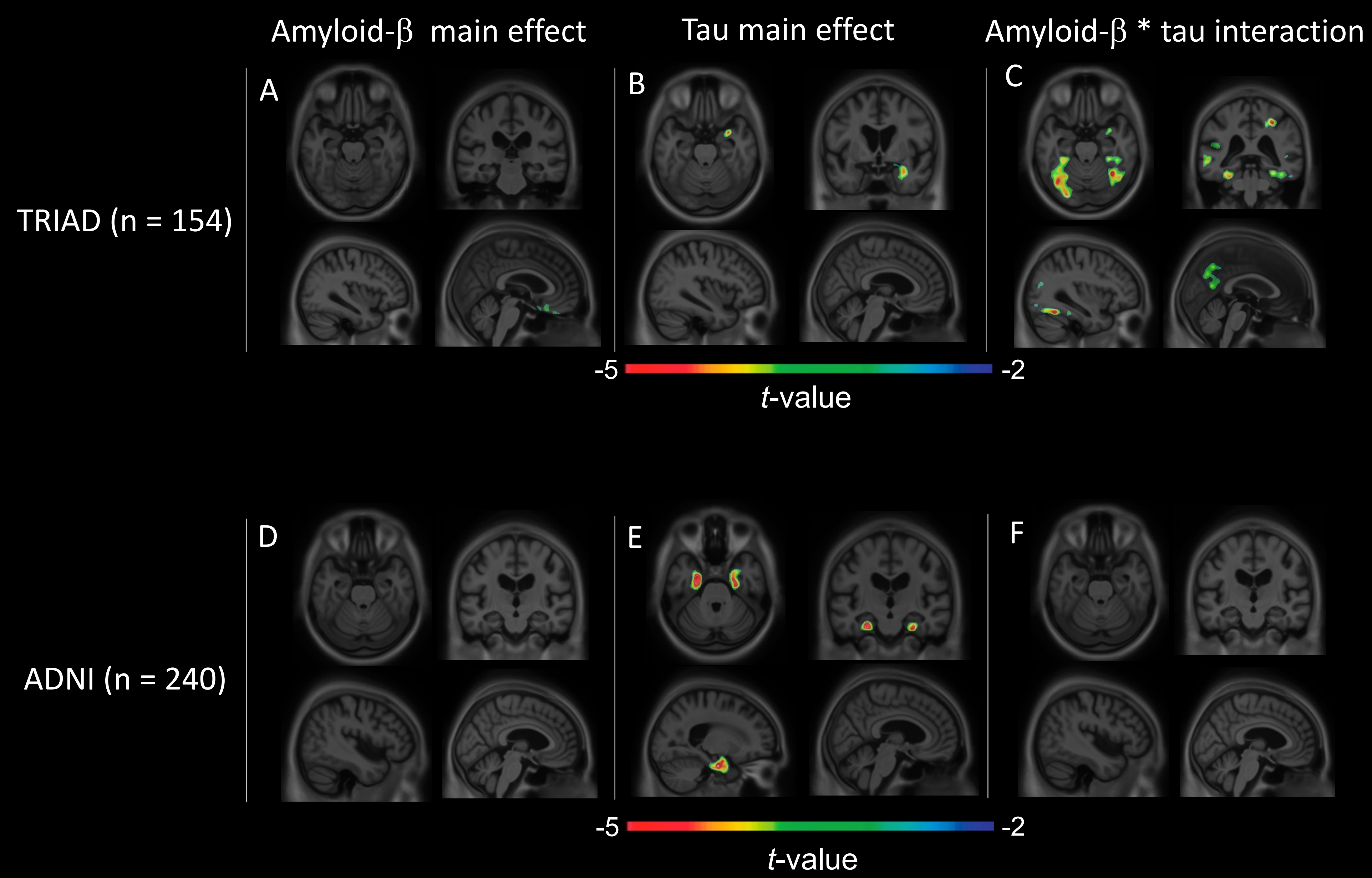
Amyloid-β main effect
Tau main effect
Amyloid-β * tau interaction
TRIAD (n = 154)
A
B
C
-5
-2
t-value
ADNI (n = 240)
D
E
F
-5
-2
t-value
