# Supplementary material for: Amyloid‐dependent and amyloid‐independent effects of Tau in individuals without dementia
Source: Ann Clin Transl Neurol. 2021 Oct 7;8(10):2083–92. doi: 10.1002/acn3.51457 (PMC8528464; doi:10.1002/acn3.51457)
Supplement: Supplementary file 5 — Table S1. Between‐sample demographic comparisons. [file ACN3-8-2083-s006.docx]

**Supplementary Table 1.** Between-sample demographic comparisons

|  | TRIAD | ADNI | *P* value | TRIAD | ADNI | *P* value |
| --- | --- | --- | --- | --- | --- | --- |
|  | CN | CN | *P* value | MCI | MCI | *P* value |
| No. | 124 | 157 | **—** | 50 | 83 | **—** |
| Age, y, mean (SD) | 70.41 (6.5) | 70.98 (5.91) | 0.77 | 70.88 (7.7) | 70.57 (7.09) | 0.81 |
| Male, no. (%) | 53 (43) | 71 (45) | 0.68 | 25 (50) | 49 (59) | 0.31 |
| Education, y, mean (SD) | 15.52 (3.86) | 16.65 (2.5) | 0.003 | 14.26 (3.79) | 15.84 (2.85) | 0.007 |
| *APOE ε4 carriers,* % | 38 (31) | 49 (31) | 0.91 | 18 (36) | 27 (32.5) | 0.01 |
| MMSE, mean (SD) | 29.05 (1.25) | 28.97 (1.33) | 0.6 | 27.13 (2.39) | 28.05 (2.15) | 0.02 |
| CDR SoB, mean (SD) | 0.18 (0.45) | 0.009 (0.51) | 0.004 | 1.47 (1.23) | 1.46 (0.93) | 0.95 |
