# Supplementary material for: Amyloid‐dependent and amyloid‐independent effects of Tau in individuals without dementia
Source: Ann Clin Transl Neurol. 2021 Oct 7;8(10):2083–92. doi: 10.1002/acn3.51457 (PMC8528464; doi:10.1002/acn3.51457)
Supplement: Supplementary file 6 [file ACN3-8-2083-s003.docx]

Supplementary Figure Legends

**Supplementary Figure 1: Regional associations between amyloid**-β**, tau and MMSE in the TRIAD and ADNI cohorts**

﻿T-statistical parametric maps were corrected for multiple comparisons using a random field theory voxel threshold of p < 0.001 and a cluster threshold of P < 0.05, overlaid on a reference template. Age, sex, and years of education were used as covariates the model. A: There was no significant main effect of [^18^F]AZD4694-PET SUVR on clinical function across the brain. B: There were main effects of [^18^F]MK6240-PET SUVR on CDR-SoB in the temporooccipital, basolateral temporal and medial temporal lobes. C: Interactions between [^18^F]AZD4694-PET SUVR and [^18^F]MK6240-PET SUVR on MMSE were observed in the precuneus, lateral temporal, inferior parietal, orbitofrontal and dorsolateral prefrontal cortices. D: There was a significant main effect of occipital [^18^F]Florbetapir SUVR on MMSE scores. E: There were main effects of [^18^F]Flortaucipir SUVR on MMSE scores in the inferior parietal and medial temporal lobes. F: Interactions between [^18^F]Florbetapir SUVR and [^18^F]Flortaucipir SUVR on Rey Auditory Verbal Learning test delayed recall scores were observed in dorsomedial prefrontal, ventromedial prefrontal, and dorsolateral prefrontal cortices.

**Supplementary Figure 2: Regional associations between amyloid**-β**, tau and RAVLT delayed recall in the TRIAD and ADNI cohorts**

﻿T-statistical parametric maps were corrected for multiple comparisons using a random field theory voxel threshold of p < 0.001 and a cluster threshold of P < 0.05, overlaid on a reference template. Age, sex, and years of education were used as covariates the model. A: There was a significant association between [^18^F]AZD4694-PET SUVR in a cluster in the medial prefrontal cortex with Rey Auditory Verbal Learning test delayed recall scores. B: There was a main effect of [^18^F]MK6240-PET SUVR on RAVLT delayed recall scores in the left medial temporal lobe. C: Interactions between [^18^F]AZD4694-PET SUVR and [^18^F]MK6240-PET SUVR on RAVLT delayed recall scores were observed in the posterior cingulate, precuneus, temporooccipital and right inferior parietal cortices. D: There was no significant main effect of [^18^F]Florbetapir SUVR on RAVLT delayed recall scores across the brain. E: There were main effects of [^18^F]Flortaucipir SUVR on Rey Auditory Verbal Learning test delayed recall scores in medial temporal lobes. F: No interactions between [^18^F]Florbetapir SUVR and [^18^F]Flortaucipir SUVR on RAVLT delayed recall scores were observed across the brain.

**Supplementary Figure 3: 3D scatter plot of the distribution of amyloid-PET and tau-PET on CDR Sum of Boxes.**

The TRIAD cohort is presented on the left, while the ADNI cohort is presented on the right. Amyloid-PET SUVR is displayed on the x axes, tau-PET SUVR is displayed on the y axes, and CDR Sum of Boxes score is displayed on z axes. Amyloid-PET and tau-PET SUVRs were extracted from the significant clusters of the interaction effects displayed in Figures 1 and 2.

**Supplementary Figure 4: 3D scatter plot of main effects of medial temporal tau-PET on CDR Sum of Boxes.**

The TRIAD cohort is presented on the left, while the ADNI cohort is presented on the right. Amyloid-PET SUVR is displayed on the x axes, tau-PET SUVR is displayed on the y axes, and CDR Sum of Boxes score is displayed on z axes. Tau-PET SUVRs were extracted from the significant clusters of the main effects of tau-PET displayed in Figures 1 and 2, while amyloid-PET SUVRs were calculated using a neocortical composite.
